# CMR in heart failure patients with left bundle branch block: pathophysiology before tissue characterization for better selection of candidates for resynchronisation therapy

**DOI:** 10.1007/s10554-021-02222-2

**Published:** 2021-03-21

**Authors:** C. Grigoratos, G. Mavraganis, G. Georgiopoulos

**Affiliations:** 1 Cardiology and Cardiovascular Medicine Department, Fondazione Toscana Gabriele Monasterio, Pisa, Italy; 2 Department of Clinical Therapeutics, National and Kapodistrian University of Athens, Athens, Greece; 3 Department of Cardiovascular Imaging, School of Biomedical Engineering and Imaging Sciences, Guy’s and St Thomas’ NHS Foundation Trust, Westminster Bridge Road, London, SE1 7EH UK

In this issue of the International Journal of Cardiovascular Imaging, Aimo et al. [1] present the results of their work on patients with non-ischaemic systolic heart failure (HF) and left bundle branch block (LBBB) undergoing cardiac magnetic resonance (CMR). They studied the clinical and prognostic significance of a wide or a narrow pattern (WP/NP) of the systolic phase of the left ventricular (LV) volume/time (V/t) curve derived from cine images. Patients classified to the WP showed more advanced HF, in terms of ventricular remodeling and neuro-ormonal activation, worse outcome and a better response to cardiac resynchronization therapy (CRT) than those with NP. Of importance, the pattern of activation, either WP or NP, did not correlate with QRS duration or with the presence and extent of myocardial scar assessed by means of late gadolinium enhancement (LGE).

LBBB is a common finding in patients with HF and an integral part in algorithms used to guide device-therapy decisions. While CRT has been established as a valuable and effective therapy in advanced HF, results from its implementation may substantially vary among patients with HF. To reduce the number of non-responders to CRT, different approaches have been proposed and are currently being applied with respect to implantation techniques and device optimization. Still, appropriate patient selection remains the cornerstone of CRT-related improvement in HF. To date, recommendations for CRT are based on morphology and duration of QRS as surrogate markers of ventricular dyssynchrony. Despite support from pathophysiologic mechanisms, imaging derived criteria have shown inconsistent results in selection of candidates for CRT and are difficult to translate into common clinical practice on grounds of complexity and heterogeneity in available software platforms. Echocardiography [2], CMR [2] and more recently CT [3] have emerged as potential tools for quantification of LV dyssynchrony in patients with HF and LBBB. Recently, Borgquist et al. [3] evaluated patients with indication for CRT applying a multimodality imaging approach. Disappointingly, the combination of echocardiography, CMR and CT for scar presence, venous anatomy and radial strain-guided LV lead placement failed to demonstrate benefit in terms of clinical or echocardiographic response and did not reduce death or HF hospitalization in this population.

The present publication represents the latest contribution in this ongoing quest to identify patients with HF and LBBB and a greater potential benefit from CRT and for that authors should be congratulated. By applying a simple yet efficient technique, Aimo et al. [1] were able to dichotomize patients with LBBB and successfully predict those with a higher likelihood of CRT response regardless of QRS duration and presence or extent of myocardial scar. Thus, V/t curves by CMR may outperform conventionally considered parameters such as LVEF, ventricular volumes and LGE in pinpointing clinically relevant LV. Finally, findings from the work from Aimo et al. [1] should serve as hypothesis generating trigger for future studies in similar scenarios such as HF patients with non-LBBB on baseline ECG. Patients with HF and non-LBBB usually have a higher rate of nonresponse after CRT implantation and current guidelines provide only class II evidence about usefulness of device therapy in this population. If more sensitive markers than ECG morphology can discriminate LV mechanical dyssynchrony, this could lead to less missed opportunities for life-prolonging device therapy. By confirming similar results with the use of volume/time curves in patients with non-LBBB, an additional brick to the wall of better understanding HF pathophysiology would be added and patients with an indication for CRT shall be better selected.
